# Prevalence and factors associated with postnatal depression among women in two rural districts of Manicaland, Zimbabwe

**DOI:** 10.4102/sajpsychiatry.v24i0.1176

**Published:** 2018-11-12

**Authors:** James January, Moses J. Chimbari

**Affiliations:** 1 Department of Psychiatry, Nelson R. Mandela School of Medicine, University of KwaZulu-Natal, South Africa; 2 School of Nursing and Public Health, College of Health Sciences, University of KwaZulu-Natal, South Africa

## Abstract

**Background:**

Low- and middle-income countries are disproportionately affected by postnatal depression (PND). High prevalence of PND in urban Zimbabwe has been reported but the situation in rural settings is largely unknown and this is one of the first studies to report prevalence of PND in Chipinge and Mutasa districts.

**Objectives:**

This study explored the prevalence and associated factors of PND among women attending postnatal care services in two rural districts of Chipinge and Mutasa, Manicaland, Zimbabwe between August and September 2017.

**Methods:**

One hundred and ninety-two women were recruited consecutively as they attended postnatal services at 7 days and 42 days post-delivery. The *Diagnostic and Statistical Manual for Mental Disorders, fifth edition* criteria was used to classify depression among participants. Prevalence of PND and 95.0% confidence intervals (CIs) were estimated and associations with key socio-demographic and risk factors assessed.

**Results:**

The mean age of participants was 23.7 years (standard deviation = 6.14). Pooled prevalence of PND across the two districts was 26.0% (95% CI: 19.04–31.74). There was a higher prevalence of PND in Mutasa (31.0%) as compared to Chipinge (21.48%) but this was not statistically significant (*p* = 0.142). Having insufficient food in the household, intimate partner violence and having a child with birthweight under 2500 g significantly increased the likelihood of PND twofold: adjusted odds ratio (aOR) = 2.8 (95% CI: 1.2–6.1), aOR = 2.5 (95% CI: 1.2–5.3) and aOR = 2.4 (95% CI: 1.1–5.6), respectively.

**Conclusion:**

The high prevalence of PND and its associated risk factors indicates the need for routine screening and targeted interventions for PND in Zimbabwe, especially in rural areas.

## Introduction

Postnatal depression (PND) is a major threat to survival of both mother and child. The World Health Organization has ranked depression as the single largest contributor to global disability, affecting 322 million people and accounting for 7.5% of years lived with disability in 2015.^1^ A recent systematic review reported an overall adjusted pooled global prevalence of perinatal depression of 11.9% in 2017.^2^ Low- and middle-income countries (LMICs) are disproportionately affected, with an estimated pooled prevalence of PND of 19.0% in 2016.^3^ Studies from sub-Saharan Africa have reported higher rates of PND exceeding 30.0% in South Africa, Nigeria and Zimbabwe,^3^ with substantial within-country heterogeneity. For instance, within Zimbabwe, PND ranged from 16.0% to 34.2% across different settings between 1995 and 2015.^4^

Several studies have highlighted strong associations between infant mortality and maternal perinatal depression.^5,6,7,8^ Given that the effects of PND transcend beyond the mother to include the family and communities,^9^ there is need to understand the factors that place postnatal women at risk of PND. Factors such as low socio-economic status and intimate partner violence have been reported to be associated with PND in most LMICs.^3^ A recent review on depression in Zimbabwean women showed that significant risk factors for PND include socio-economic difficulties, history of experiencing adverse events and intimate partner violence.^4^

Research on the prevalence and factors associated with antenatal and PND in Zimbabwe has largely focused on women in urban areas.^10,11,12,13^ However, women in rural areas may be at greater risk of PND than their urban counterparts. For instance, a study conducted in rural areas of South Africa reported a 50.3% prevalence of PND^14^ against 39.5% in urban areas.^15^ This indicates the need to explore whether Zimbabwean women living in rural settings are equally affected as their urban counterparts. Women in rural areas may also have less access to and low quality of mental healthcare as compared to those in urban areas. Furthermore, most studies that have measured perinatal depression in Zimbabwe used screening tools such as the Centre for Epidemiological Studies-Depression (CES-D),^13^ the Edinburgh Postnatal Depression Scale (EPDS)^12,16^ and the Shona Symptom Questionnaire.^17,18^ Use of screening tools to ascertain depressive symptoms provides mental health practitioners and researchers with useful data in resource-limited settings. However, relying solely on results from these surveys presents challenges including non-use of uniform cut-off scores, which can result in overestimation or underestimation of PND among women.^4^ This indicates the need for studies utilising gold standard diagnostic tools such as the Structured Clinical Interview of the Diagnostic Statistical Manual for Mental Disorders, Fifth Edition (SCID-5)^19^ for estimating the burden of perinatal depression. Thus, this study aimed to determine the prevalence and factors associated with PND using the Diagnostic and Statistical Manual for Mental Disorders, fifth edition (DSM-5) criteria among postnatal care attendees in two rural districts of Zimbabwe.

## Methods

### Design and setting

Details of how this study was conducted have been described in an earlier publication.^20^ An analytic cross-sectional study was conducted among women at rural health centres in Mutasa and Chipinge districts in Manicaland Province of Zimbabwe between August and September 2017. Manicaland was purposively selected as it had the highest under-5 mortality, the lowest percentage of antenatal care from a skilled provider (86.4%) and the second highest percentage of women reporting sexual violence (16.0%) in 2015.^21^ Chipinge and Mutasa districts were purposively selected out of the seven districts in the province as they had the lowest and highest postnatal care coverage in the province, respectively, according to the Multiple Indicator Cluster Survey of 2014. The three major hospitals in Mutasa, namely Bonda Mission Hospital, Hauna District Hospital and Old Mutare Hospital were purposively included in the study. In Chipinge, two major hospitals (Chipinge District Hospital and Mount Selinda Mission Hospital) were included.

### Participants

A total of 453 women were enrolled into the study and responded to questionnaires. However, only 192 were assessed by the clinical psychologist using the interview based on DSM-5 criteria as a result of limited resources, specifically time and qualified assessors. This paper reports results from the 192 women who were assessed for depression using the DSM-5 criteria. The screening instruments which were administered to all the 453 women included the EPDS, the Patient Health Questionnaire-9 and CES-D. Sample size for the validation study was determined using the Dobson formula, assuming a prevalence of PND among women in rural areas to be 50.0% (assumes maximum variability and yields largest sample size) as there are no studies that have estimated the prevalence for this population in Zimbabwe.^20^ Furthermore, the assumed 50.0% prevalence resonates well with the 50.3% reported among rural women in South Africa.^14^ This yielded a sample size of 385, which was adjusted to 462 assuming a 20.0% non-response rate.

### Procedures

Participants were recruited consecutively as they attended postnatal services at 7 days and 42 days post-delivery at postnatal clinics in the two districts. Three trained research assistants, with degree-level education, individually approached potential participants and after obtaining written consent, collecting socio-demographic information and administering three depression screening tools referred the women to a clinical psychologist for administration of the DSM-5 interview. Socio-demographic data were collected and entered into an Android-based mobile application (Open Data Kit) and harmonised with data collected using the DSM-5 interview in Microsoft Office Excel package. Inclusion criteria for participation included being a mother attending postnatal care at 7 days and 6 weeks post-delivery and we excluded women with psychosis, those admitted as inpatients and those who were bedridden and unable to freely give written consent. Those with severe depression were included. Data quality checks were performed in the field where we checked for completeness and consistency of responses.

### Measures

A clinical psychologist conducted structured clinical interviews to assess participants for the presence of PND using DSM-5 criteria for depression. Diagnosis of current major depression used the DSM-5 criteria, whereby five or more of the following symptoms have to be present during a 2-week period: (1) depressed mood most of the day nearly every day, (2) anhedonia or markedly decreased interest or loss of interest or pleasure in almost all activities, (3) clinically significant weight loss or increase or decrease in appetite, (4) insomnia or hypersomnia, (5) psychomotor agitation or retardation, (6) fatigue or loss of energy, (7) feelings of worthlessness or excessive or inappropriate guilt, (8) diminished ability to think or concentrate, or indecisiveness and (9) recurrent thoughts of death or suicidal ideation. The DSM-5 criteria further stipulates that at least one of these symptoms should be either (a) a depressed mood or (b) loss of interest or pleasure or anhedonia.^22^ Although the DSM criteria for major depressive disorder with peri-partum onset is defined as up to 4 weeks post-delivery, in this study we considered women up to 6 weeks post-delivery as the scheduled postnatal visits in Zimbabwe are at 7 days and 6 weeks.^20^

### Data analysis

The data were analysed using STATA version 14.^23^ Bivariate and multivariable analyses were performed to identify factors associated with PND. The Pearson chi-square (χ^2^) test or Fisher’s exact test were performed to assess bivariate associations. Bivariate and multivariable logistic regression were performed to identify factors associated with PND. Coefficients were exponentiated to present odds ratios (ORs) and their 95% confidence intervals (CIs). Statistical significance for all tests was set at an alpha level of 0.05.

### Results

The mean age of the respondents was 23.7 years (standard deviation = 6.14). The majority (*n* = 166, 86%) of the women were aged between 18 and 30 years. Most (*n* = 136, 71%) women in our sample had attained secondary school education. Table 1 summarises socio-demographic characteristics of respondents screened using the DSM-5 criteria.

**TABLE 1:** Demographic characteristics of study participants (*N* = 192).

Description	Frequency
**Age**	
Mean (s.d.)	23.73 (6.14)
Range	16–40
Interquartile range	19.5–27
**Marital status, *n* (%)**	
Married	121 (63.0)
Single	17 (8.9)
Cohabiting	46 (23.9)
Divorced	7 (3.6)
Widowed	1 (0.5)
**Highest level of education, *n* (%)**	
Primary	48 (25.0)
Secondary	121(63.0)
Tertiary	15 (7.8)
Never been to school	8 (4.2)

s.d., standard deviation.

## Ethical consideration

Permission to conduct the study was granted by the Zimbabwean Ministry of Health and Child Care at national, provincial, district and facility levels. The research protocols were reviewed and approved by the institutional review boards of the University of KwaZulu-Natal Biomedical Research Ethics Committee (BE598/16), the Joint Research Ethics Committee for the University of Zimbabwe, College of Health Sciences and the Parirenyatwa Group of Hospitals (JREC/30/17) and the Medical Research Council of Zimbabwe (MRCZ/A/2161). All the women who experienced distress during the study were referred for appropriate psychological care within their district. Explanations were given to women on potential of the interview to evoke emotions and their right to terminate the interview at any stage without penalty or prejudice. Because there were no psychiatrists and/or clinical psychologists in the two districts, all women diagnosed with severe depression or seen to harbour suicidal thoughts were referred to psychiatric nurses stationed at the hospitals where the women sought postnatal services.

## Prevalence of depression

The pooled prevalence of PND in both districts was 26% (95% CI: 19.04–31.74) with women in Mutasa having a higher prevalence of 31% compared to those in Chipinge (21.48%). However, the difference in the prevalence across districts was not statistically significant (*p* = 0.142). Women who reported having insufficient food in the last month (*p* = 0.005), those who could not afford to send their children to school (*p* = 0.014), those who reported community violence (*p* = 0.009), those who had no social support (*p* = 0.004) and those who did not have access to health information (*p* = 0.009) had statistically significant higher prevalence of PND.

Differences in prevalence of depression across categories are presented in Table 2.

**TABLE 2:** Prevalence of postnatal depression by category among participants (*N* = 192).

Variable	Depression				p*
	Not depressed		Depression		
	*n*	%	*n*	%
Total sample	144	75.00	48	25.00	
**District**					
Mutasa	49	69.01	22	30.99	0.142
Chipinge	95	78.51	26	21.48	
**Marital status**					
Married	96	79.34	25	20.66	0.144
Single	13	76.47	4	23.53	
Cohabiting	31	67.39	15	30.61	
Divorced or separated	3	42.86	4	57.14	
Widowed	1	100.00	0	0.00	
**Age (years)**					
16–20	55	73.33	20	26.67	0.132
21–25	45	77.59	13	22.51	
26–30	25	73.53	9	26.47	
31–35	12	85.71	2	14.29	
36–40	7	63.64	4	36.36	
**Education**					
Primary	40	71.43	16	28.57	0.87
Secondary	92	76.03	29	23.00	
Tertiary	12	80.00	3	20.00	
**No food in last month**					
Yes	16	55.17	13	44.83	0.005**
No	123	79.35	32	20.65	
**Affording to send children to school**					
Yes	113	79.58	29	20.42	0.014**
No	31	62.00	19	38.00	
**Access to health information**					
Yes	119	79.33	31	20.66	0.009**
No	25	59.52	17	40.48	
**Number of pregnancies in lifetime**					
1	50	72.46	19	27.54	0.039**
2	47	88.68	6	11.32	
3	21	61.76	13	38.24	
4	10	66.67	5	33.33	
**Sex of child**					
Male	70	74.47	24	25.53	0.868
Female	74	75.51	24	24.49	
**Presence of community violence**					
Yes	45	64.29	25	35.71	0.009**
No	99	81.15	23	18.85	
**Self-reported intimate partner violence in last 12 months**					
Yes	56	62.92	33	37.08	0.001**
No	86	85.15	15	14.85	
**Mother’s self-reported HIV status**					
Positive	44	62.86	26	37.14	0.003**
Negative	100	81.97	22	18.03	
**Self-reported presence of social support**					
Yes	111	80.43	27	19.57	0.004**
No	32	60.38	21	39.62	

* Chi-squared (χ^2^) test or Fisher’s exact test used to compare categorical variables;

** Statistically significant.

## Factors associated with postnatal depression

There was no statistically significant association between age and PND (*p* = 0.750). Based on the bivariate analysis: not having enough food in the household (OR = 2.9, 95% CI: 1.4–6.1), experiencing intimate partner violence (*OR* = 3.4, 95% CI: 1.72–6.94) and being HIV positive (OR = 2.7, 95% CI: 1.4–5.2) were significantly associated with PND (Table 3). Women who reported educational level below primary level were significantly less likely to be depressed compared to women who had reached secondary level of education (OR = 0.15, 95% CI: 0.08–0.31). After multivariable adjustment, women who reported not having enough food in the household were 2.8 times more likely to experience PND (adjusted odds ratio 2.8, 95% CI: 1.2–6.1), while those who experienced intimate partner violence were more than twice as likely to be depressed (adjusted odds ratio [aOR] = 2.5, 95% CI: 1.2–5.3). Having a child who was below 2500 g at birth also remained statistically significantly associated with PND (aOR = 2.4, 95% CI: 1.1–5.6).

**TABLE 3:** Factors associated with postnatal depression among study participants (*N* = 192).

Variable	Bivariate			Adjusted		
	OR	95% CI	*p*	OR	95% CI	*p*
Inadequate food in household	2.9	1.4–6.1	0.005**	2.8	1.2–6.1	0.013
Self-reported intimate partner violence	3.4	1.7–6.9	0.001**	2.5	1.2–5.3	0.017
	2.4	1.1–5.1	0.024**	2.4	1.1–5.6	0.038
Mother self-reporting as HIV positive	2.7	1.4–5.2	0.004**	1.8	0.3–2.8	0.114

OR, odds ratio; CI, confidence interval.

** Statistically significant.

## Discussion

This study assessed the prevalence and factors associated with PND among women attending post-delivery services in two rural districts of Zimbabwe. It is one of the first studies to report prevalence of PND in Chipinge and Mutasa districts. The prevalence of PND among women was high with no statistically significant differences between the districts. Prominent risk factors for PND that were identified included not having enough food to eat within the household, having a child with low birthweight and experiencing intimate partner violence.

Elevated prevalence of PND was observed among women attending postnatal care services in rural Manicaland, confirming trends highlighted in previous studies. For instance, similar prevalence of PND (21.4%) was found in urban areas of Zimbabwe during pregnancy^13^ and in the postnatal period (30.0% – 34.0%).^11,12,16^ Contrary to our findings, a study in rural South Africa reported the prevalence of PND among women to be 50.3%.^14^ The discrepancies could be because of differences in settings and in the instruments used with the South African study having used the EPDS and Beck Depression Inventory. The high prevalence of PND in our sample underscores the need for instituting appropriate interventions.

Women in our study who reported that they lacked enough food in the household were more likely to be depressed. Previous studies from Zimbabwe have also implicated socio-economic factors such as unemployment as predisposing women to depression.^4,11^ Our results also indicated that low birthweight of the baby was associated with depression, which confirms findings from other settings.^24^ Women who have babies with low birthweight may be worried about the health of their child, which may predispose them to depression. It is also plausible to assume that these women may have been depressed during pregnancy, which may have predisposed their children to low birthweight. Our findings support the view that intimate partner violence increases the odds of depression among women during the perinatal period. Results from other settings have also confirmed this.^12,13,25,26,27^ Thus, there is need to identify women who may be experiencing intimate partner violence so that timely interventions may be instituted. Interventions that promote community awareness of the negative impacts of intimate partner violence need to be fostered as there is evidence suggesting that such efforts are effective in reducing and preventing violence in low-resourced settings.^28^ These factors tend to support the notion that there could be an interplay of common socio-economic factors, which might be predisposing women to PND.

Limitations of our study include the smaller sample size comprising voluntary participants that potentially introduced bias as women with depressive symptoms may have refused to participate. The sample size might have resulted in some factors not being statistically significant. Additionally, the study was conducted in hospitals, which makes it difficult to generalise results to the community. The cross-sectional nature of the study meant that all women were assessed for PND only once and future studies could adopt a longitudinal approach so as to ascertain whether some women assessed at 7 days would develop PND later on. Lastly, diagnosis of depression was determined by only one clinical psychologist making it impossible to assess interviewer bias. These limitations need to be addressed in future studies, particularly the use of larger sample sizes and having multiple assessors. Notwithstanding this, it is crucial not to overlook that this is one of the first studies in Zimbabwe to explore the burden of PND among rural populations. Thus, we have provided the base upon which future studies can build on in exploring antecedents of PND among women.

## Conclusion

We conclude that PND among women in two rural districts of Manicaland, Zimbabwe, is high and warrants intervention. Risk factors for PND included not having enough food, experiencing intimate partner violence and having a baby who was underweight. Our findings are important for considering the management of women with depression during the perinatal period. We recommend that primary healthcare providers should screen for and offer interventions for depression as a routine component of postnatal care.
